# A Scoping Review on Barriers to Mental Healthcare in Canada as Identified by Healthcare Providers

**DOI:** 10.1192/bjo.2022.258

**Published:** 2022-06-20

**Authors:** Jeffrey Wang, Stanislav Pasyk, Claire Slavin-Stewart, Andrew Olagunju

**Affiliations:** 1 McMaster University, Hamilton, Canada; 2 St Joseph's Healthcare Hamilton, Hamilton, Canada

## Abstract

**Aims:**

Mental illness is among the leading causes of disability globally, however the treatment gap is wide even for developed countries. The perspectives of patients and mental healthcare providers are critical to understanding barriers to adequate mental healthcare and developing scalable solutions that improve access and quality of services. However, the views of providers are relatively understudied, precipitating our review to collate and synthesize their perspectives on the barriers to mental healthcare in Canada.

**Methods:**

We searched MEDLINE/PubMed and PsychINFO for studies with findings in Canada published in English from 2000–2021 with terms for mental health, psychiatry, barriers, and referrals. Included studies were evaluated with the National Institutes of Health Study Quality Assessment Tools and Critical Appraisal Skills Programme.

**Results:**

631 papers were screened, finding 20 eligible studies, including 13 qualitative, one cross-sectional, one retrospective, and five mixed-methods studies. Through inductive content analysis, five themes of barriers emerged: (1) patient accessibility (19% of studies), (2) health systems availability and complexity (31%), (3) training/education (25%), (4) work conditions (21%), and (5) cultural sensitivity (4%). Among barriers discussed, common challenges included a lack of resources for both patients and providers, gaps in continuing education for primary care providers, and health systems challenges such as difficulty securing referrals, unclear intake criteria, and confusion due to overload of contacts.

**Conclusion:**

Health systems face a multi-faceted set of challenges to improving access to mental healthcare that will require solutions from various stakeholders. Understanding these barriers is critical in focusing initiatives to improve mental health care, both in Canada and in countries facing similar challenges.

